# RT-RPA-*Pf*Ago detection platform for one-tube simultaneous typing diagnosis of human respiratory syncytial virus

**DOI:** 10.3389/fcimb.2024.1419949

**Published:** 2024-07-25

**Authors:** Jia-Yu Liao, Xue-Yong Feng, Jie-Xiu Zhang, Tian-Dan Yang, Min-Xuan Zhan, Yong-Mei Zeng, Wei-Yi Huang, Hao-Bin Lian, Lin Ke, Si-Si Cai, Nan-Fei Zhang, Jin-Wen Fang, Xiao-Ying Cai, Jun-Duo Chen, Guang-Yu Lin, Li-Yun Lin, Wei-Zhong Chen, Yu-Yan Liu, Fei-Fei Huang, Chuang-Xing Lin, Min Lin

**Affiliations:** ^1^ Department of Pediatrics, Second Affiliated Hospital of Shantou University Medical College, Shantou, Guangdong, China; ^2^ Shantou University Medical College, Shantou, Guangdong, China; ^3^ School of Food Engineering and Biotechnology, Hanshan Normal University, Chaozhou, Guangdong, China; ^4^ Department of Medical Laboratory, Chaozhou People’s Hospital Affiliated to Shantou University Medical College, Chaozhou, Guangdong, China; ^5^ School of Laboratory Medicine, Youjiang Medical University for Nationalities, Baise, Guangxi, China

**Keywords:** human respiratory syncytial virus (HRSV), typing diagnosis, reverse transcription recombinase polymerase amplification (RT-RPA), pyrococcus furiosus argonaute, point of care (POC)

## Abstract

Human respiratory syncytial virus (HRSV) is the most prevalent pathogen contributing to acute respiratory tract infections (ARTI) in infants and young children and can lead to significant financial and medical costs. Here, we developed a simultaneous, dual-gene and ultrasensitive detection system for typing HRSV within 60 minutes that needs only minimum laboratory support. Briefly, multiplex integrating reverse transcription-recombinase polymerase amplification (RT-RPA) was performed with viral RNA extracted from nasopharyngeal swabs as a template for the amplification of the specific regions of subtypes A (HRSV_A_) and B (HRSV_B_) of HRSV. Next, the *Pyrococcus furiosus Argonaute* (*PfAgo*) protein utilizes small 5’-phosphorylated DNA guides to cleave target sequences and produce fluorophore signals (FAM and ROX). Compared with the traditional gold standard (RT-qPCR) and direct immunofluorescence assay (DFA), this method has the additional advantages of easy operation, efficiency and sensitivity, with a limit of detection (LOD) of 1 copy/μL. In terms of clinical sample validation, the diagnostic accuracy of the method for determining the HRSV_A_ and HRSV_B_ infection was greater than 95%. This technique provides a reliable point-of-care (POC) testing for the diagnosis of HRSV-induced ARTI in children and for outbreak management, especially in resource-limited settings.

## 1 Introduction

The most common infectious illness in pediatrics is acute respiratory tract infection, which can be classified as acute upper respiratory tract infection (AURTI) and acute lower respiratory tract infection (ALRTI). Moreover, ALTRI is a leading etiology of mortality in children under the age of five after infection (Nair et al., 2010). Viruses, bacteria, atypical pathogens, and fungi are among the pathogens that cause respiratory tract infections. Among these pathogens, human respiratory syncytial virus (HRSV) is the most common viral infection causing ALRTI in children under the age of five around the world (Kyu et al., 2016). HRSV is a virus of the *Paramyxoviridae* family that is enveloped and nonsegmented, with a single negative-strand RNA genome (Collins et al., 2013). HRSV can be divided into two antigenic subgroups, HRSV_A_ and HRSV_B_, which exhibit genome-wide sequence divergence. Both of these subtypes are widespread in the population, with subtype A being more common (Peret et al., 2000; Ren et al., 2015; Song et al., 2017). Determining the diversity and frequency of its genome can be supported by HRSV typing. Research on the epidemiological characteristics of HRSV vaccine development and clinical principles is significantly facilitated by this categorization (Borchers et al., 2013; Papi et al., 2023; Pierangeli et al., 2023).

According to epidemiological studies, HRSV-infected children accounted for 22% of all ALTRI-infected children between 2013 to 2022, with approximately 200,000 deaths accounting for 9% of the total deaths (Kyu et al., 2016). Accurate and timely diagnosis of HRSV infection is undoubtedly beneficial for patient care and reducing unnecessary antibiotic utilization. This approach may also minimize ancillary testing, decrease the length of hospitalization, and make it possible for isolation to be quickly implemented to stop the transmission of viruses in hospitals and long-term care settings (Chartrand et al., 2015). Therefore, rapid and sensitive point-of-care (POC) testing is vital for the clinical diagnosis and treatment of HRSV infection. Currently, there are three HRSV diagnostic modalities in clinic. Viral culture is the gold standard for traditional HRSV diagnosis, but it has a long turnaround time, limited sensitivity and susceptibility to multiple factors. Direct immunofluorescence assay (DFA) and rapid antigen detection tests (RADTs) for HRSV detection are widely used and commercialized methods but are less sensitive than PCR (Chartrand et al., 2015). Reverse transcriptase PCR (RT-PCR) has a much shorter turnaround time, high sensitivity and specificity (Falsey and Walsh, 2006), but it requires specific thermal cyclers and complex, time-consuming operation (Banerjee et al., 2019). All these factors make its implementation challenging in resource-limited countries. Therefore, it is urgent to develop a point-of-care (POC) testing for HRSV infection.

To address the demand for POC testing, isothermal nucleic acid amplification methods have been introduced (Liu et al., 2023). These methods included recombinase polymerase amplification (RPA), recombinase-aided amplification (RAA), multiple-cross-displacement amplification (MCDA) and loop-mediated isothermal amplification (LAMP). Compared with typical PCR methods, these tools can be applied in 30–40 minutes at a constant temperature using a single-temperature heat source (e.g., a water bath) without the need for commercial thermocyclers (Nam and Ison, 2019; Qian et al., 2021). These advantages also enable them to develop a suitable POC testing in this field, especially in low-resource settings. RPA has undergone rapid development in recent years as an isothermal amplification technique due to its high speed, sensitivity, multiplexibility, and constant temperature reaction conditions. Therefore, we can construct new nucleic acid detection techniques with rapidity, accuracy, and high sensitivity based on these isothermal amplification techniques. Recently, the molecular diagnostic community has been intrigued by programmable endonucleases, such as *prokaryotic Argonaute* (*pAgo*) and CRISPR/Cas (Clustered Regularly-Interspaced Short Palindromic Repeats). *Pyrococcus furiosus Argonaute* (*PfAgo*) is a novel DNA-guided programmable endonuclease. It utilizes small 5’-phosphorylated single-strand DNA guides to specifically cleave DNA targets without the need for a protospacer-adjacent motif (PAM) or protospacer flanking site (PFS) on the target sequence, which largely extends its application in the selection of available target DNA sequences (Swarts et al., 2015; Enghiad and Zhao, 2017). In addition, unlike CRISPR/Cas systems, the *PfAgo* endonuclease exploits guide DNA (gDNA) rather than guide RNA to help target DNA cleavage. Compared to guide RNA, gDNA is more convenient for *in vitro* applications due to its greater stability and ease of synthesis. Theoretically, *PfAgo* combined with isothermal amplification technology can create highly precise, sensitive, and dependable detection assays (Kuzmenko et al., 2019). Examples included *PfAgo*-triggered detection of pathogens that infect human or animals, such as *SARS-CoV-2*(F. Wang et al., 2021; Xun et al., 2021), *human papillomavirus* (HPV) (Wang et al., 2021) and *enterocytozoon hepatopenaei* (EHP) (Yang et al., 2023).

Here, we describe a novel nucleic acid detection system that uses efficient integrated multiplex reverse transcription RPA (RT-RPA) and *PfAgo* for one-tube simultaneous typing diagnosis of HRSV_A_ and HRSV_B_. The entire detection process was completed within 60 minutes, achieving a sensitivity of 1 copy/μL. The RT-RPA-*PfAgo* reactions were carried out at constant temperatures of 39°C and 95°C, therefore a water or metal bath may suit the experimental conditions. The fluorescence signal produced by the reaction might be measured with a portable fluorescence detector or seen with the naked eye under blue light. The RT-RPA-*PfAgo* platform established in this study may have excellent application value for POC testing of HRSV infection.

## 2 Experimental section

### 2.1 Materials

All primers, gDNA, probes and HRSV standards were synthesized by GENEWIZ Biotechnology Co., Ltd. (Suzhou, China). The basic RPA kit was purchased from Qitian Gene Biotechnology Co., Ltd. (Jiangsu, China). The purification kit was obtained from Tiangen Biochemical Technology (Beijing, China).

### 2.2 Nucleic acid preparation

The RPA primers were designed using parameters according to the TwistAmp® Analytical Design Manual (https://www.twistdx.co.uk/support/rpa-assay-design/) and BLAST(Basic Local Alignment Search Tool, National Center for Biotechnology Information) in combination with Primer Premier 5.0 software (Premier Biosoft, San Francisco, CA, USA). Alignment analysis of five HRSV subtype A strains (NCBI Accession: KT992094.1, MW020599.1, MZ221205.1, ON729318.1, and KY967364.1) and four HRSV subtype B strains (NCBI Accession: MG642066.1, MF001058.1, MH327947.1, and OM857397.1) was performed to evaluate the sequence specificity of the primers. Within the respective range of the best primer pair amplification, two pairs of gDNA were designed on the righteous strand and antisense strand, and their corresponding probes were designed on this basis. All primers, gDNA, and fluorescent probes were synthesized by GENEWIZ (GENEWIZ Biotechnology Co., Ltd., Suzhou, China). All oligonucleotides are shown in **Table 1**.

**Table 1:** Oligonucleotides primers.

Oligonucleotides	Sequence (5’-3’)	Production (bp)	Location (Seq No.)
HRSV_A_F1	AGGACCTACATCTGCTCGGGATGGTA	338	10~35
HRSV_A_R1	ATTCGTTTGGTTGGATGATTGGGTTG		322~347
HRSV_A_F2	AGGACCTACATCTGCTCGGGATGGTA	330	10~35
HRSV_A_R2	GGTTGGATGATTGGGTTGGTTAGTTTG		313~339
HRSV_A_F3	CACATTAGTAGTGGCAAGTGCAGGACC	335	1~27
HRSV_A_R3	GGATGATTGGGTTGGTTAGTTTGTTGG		309~335
HRSV_B_F1	AAGACTTAGGAATGAGGAAAGCGAAAA	223	10~36
HRSV_B_R1	TTATTTGTTCGGTCTGGCAGTTGATTC		206~232
HRSV_B_F2	GCTATGGCAAGACTTAGGAATGAGG	229	2~26
HRSV_B_R2	TGGTGGTTCTGCTGACGGACGTTTA		206~230
HRSV_B_F3	AGGCTATGGCAAGACTTAGGAATGAGG	230	1~27
HRSV_B_R3	ATTTGTTCGGTCTGGCAGTTGATTC		207~231
gDNA1	P-CGCTCGGGATGGTATA		
gDNA2	P-CGAGATGCCATGGTTG		
PorbeA1	BHQ1-CCTTCGGTATAAGAGATGCCAGCAAATCAA-FAM		
gDNA3	P-CTGTAGACGAGCCCTA		
gDNA4	P-TCATATTCTCTACGGT		
PorbeA2	BHQ1-TTACCGCCCTACCATATTCTCCTTCTCCAG-FAM		
gDNA5	P-CGACAACGATAGTGAC		
gDNA6	P-CGAGTGACTTGTTGGA		
PorbeB1	BHQ2-TGACTGACCGTTGGAAGACAACGATCTGAC-ROX		
gDNA7	P-CGTGAGTTGATCACTG		
gDNA8	P-GTGTTGTTGATTGCTG		
PorbeB2	BHQ2-CACGACCTGTTGCTGAGTGAGTTGAGACAC-ROX		

### 2.3 Expression and purification of the PfAgo protein

The purification of the *PfAgo* protein (NCBI-Protein ID: WP_011011654.1) was performed following a protocol reported previously (Ye et al., 2022). In brief, codon optimization of the *PfAgo* gene was performed using JCat software (http://www.jcat.de/). The *PfAgo* gene was subsequently obtained by gene synthesis and cloned and inserted into the pET28a (+) plasmid for recombinant protein expression. Finally, the N-terminally tagged His fusion protein was induced in the *E. coli* BL21 (DE3) strain with 1 mM IPTG at 37°C and purified through Ni-affinity chromatography on an AKTA Prime Plus system (GE Healthcare Life Sciences, Boston, MA). Storage buffer (20 mM Tris-HCl (pH 8.0), 300 mM NaCl, 0.5 mM MnCl_2_, and 15% (v/v) glycerol) was used to maintain the purity of the protein, and aliquots were stored at -80°C.

### 2.4 Generation of HRSV RNA standards

The partial sequences of the HRSV subtype A strains (nt2817–3146, KT992094.1) and HRSV subtype B strains (nt2853–3107, MG642066.1) were integrated into the pGEM-T vector and transformed into Top10 competent cells by GENEWIZ (GENEWIZ Biotechnology Co., Ltd., Suzhou, China). Then, the positive inserts were transcribed using T7 RiboMAX™ Large Scale RNA Production Systems (Promega, US). The transcribed RNA concentration was determined by NanoDrop™ ND-2000 spectrophotometry (Thermo Fisher) and calculated by the formula: Amount (RNA copies/µl) = (X g/µL RNA × 6.022 ×10^23^)/(nt transcript length × 340) (Xu et al., 2020). The obtained HRSV_A_ and HRSV_B_ RNA standards were confirmed by PCR and stored at -80°C until the experiment.

### 2.5 Basic RPA and RT-RPA

Basic RPA was used for the screening of the RPA primers. The RPA assay was conducted using a commercial basic RPA kit (No: B00000) (Jiangsu Qitian Gene Biotechnology Co., Ltd., China). The 50 μL final reaction mixture contained 2.4 μL each forward primer (10 μM), 2.0 μL MgACO (280 mM), 25 μL buffer A, 2.0 μL HRSV genomic cDNA, lyophilized enzyme pellets and ddH_2_O. The reaction tube was turned up and down 10 times to mix well. The reaction time and temperature were optimized to 30 minutes and 39°C, respectively. RPA was carried out on a Life Touch Thermal Cycler (Bioer Technology Co., Ltd., China), after which all the RPA products were analyzed on a 2% agarose gel. The RT-RPA reactions were carried out according to the instructions of the RT-RPA Nucleic Acid Amplification Reagent (No: B00R00) (Jiangsu Qitian Gene Biotechnology Co., Ltd., China), and all the operations were performed according to the instruction manual. The 50 μL reaction mixture included 25 μL Buffer V, 2 μL each primer (10 μM), 4 μL RNA template, MgACO (280 mM), lyophilized enzyme pellets and ddH_2_O to replenish the remaining space.

### 2.6 PfAgo cleavage system

The 25μL *PfAgo* reaction system contained 4 μL *PfAgo* (200 U/μL), 4 μL each gDNA (10 μM), 2μL of each signal-producing ssDNA reporter (10 μM) and 2μL endonuclease reaction buffer (15 mM Tris/HCl pH 8.0, 250 mM NaCl, and 0.5 mM MnCl_2_),4 μL purified amplification product, 9μL ddH_2_O, and then incubated for 30 min at 95°C in SLAN-96S Real Time PCR Detection System (Shanghai Hongshi Medical Technology Co., Ltd, Shanghai, China) with the FAM/ROX fluorescence signal recorded in 30 seconds intervals.

### 2.7 Validation of the RT-RPA-PfAgo detection platform products

The products obtained after *PfAgo* cleavage were verified using Urea-PAGE Gel. The Urea-PAGE mixture consisted of three layers: the lowest layer was 15% Urea-PAGE (15% Urea-PAGE Gel solution 2.5 mL, 10% APS 12.5 μL, and TEMED 2.5 μL); the middle layer was 12% Urea-PAGE (12% Urea-PAGE Gel solution 4.0 mL, 10% APS 20.0 μL, and TEMED 4.0 μL); and the top layer was a concentrated gel (Urea 1.47 g, PAA 350 μL, 5% TBE 700 μL, APS 35 μL and TEMED 3.5 μL, with ddH_2_O added to 3.5 mL). Urea-PAGE was performed at 200 V for 30 minutes before use, and the sample was loaded at 200 V for 15 minutes and 150 V for 30 minutes. After electrophoresis, the Urea-PAGE gel was decolorized in staining solution for 30 mins and imaged.

### 2.8 Optimization of the RT-RPA-PfAgo detection platform

To determine the effect of each parameter on the efficiency of the *PfAgo* system, we optimized the volume of *PfAgo*, gDNA, Mn^2+^, and template to obtain the most efficient reaction system. The results of each optimization were analyzed by the SLAN-96S Real Time PCR Detection System. The reaction was carried out at 95°C for 30 minutes, and the fluorescence values were collected at 30 seconds intervals. The optimum scenario occurs when the fluorescence peaks are faster and higher.

### 2.9 Evaluation of the RT-RPA-PfAgo detection platform

To evaluate the limit of detection (LOD) of the RT-RPA-*PfAgo* platform, the synthesized HRSV RNA standards were diluted in ddH2O (ranging from 10^6^ to 1 copy/μL) and assessed by the RT-RPA-*PfAgo* platform at 39°C for 30 minutes. For specificity assessment, *SARS-CoV-2*, *influenza virus*, *adenovirus*, *Klebsiella pneumoniae*, *Streptococcus pneumoniae* and *Staphylococcus aureus* were collected. All the experiments were independently replicated three times.

### 2.10 Clinical sample preparation

A total of 150 nasopharyngeal swabs were obtained from children with respiratory illness who were hospitalized at the Children’s Hospital of the Second Affiliated Hospital of Shantou University School of Medicine, Shantou, China, between October 2022 and June 2023. The ages ranged from 1 month to 2 years. All nasopharyngeal swabs were placed in 2 ml of Dulbecco’s Modified Eagle Medium (DMEM) and stored in an ultralow temperature freezer at -80°C. This study was approved by the Medical Ethics Committee of the Second Affiliated Hospital of Shantou University Medical College (permit number: 2020–31). RNA was collected from pharyngeal swabs using an RNA Swab Extraction Kit (Solarbio Science & Technology Co., Ltd., Beijing. China) according to the reagent vendor’s instructions and stored in a -80°C freezer. All samples were tested RT-RPA-*PfAgo* platform, RT-PCR (human Respiratory Syncytial Virus (HRSV) Typing A&B Real Time RT-PCR Kit, Liferiver Bio-Tech Co., Ltd., Shanghai, China) and DFA (D^3^ Ultra DFA Respiratory Virus Screening & ID Kit, Head Biotechnology Co., Ltd., Beijing, China). The RT-PCR and DFA methods were carried out in accordance with the experiment’s instructions. In RT-PCR, a Ct value lower than that of the positive control indicates the presence of an infection with HRSV_A_ or HRSV_B_ in the sample. Under 200x magnification in a fluorescence microscope, if at least two green fluorescent cells were found in each visual field, the image was considered positive; otherwise, the direct immunofluorescence assay (DFA) was negative.

## 3 Data analysis

The data were entered into the Excel program (Microsoft Excel Home and Student 2019). The statistical analysis was performed with SPSS 25.0, and the results were tested by the t-test for the comparison of the fluorescent intensity and the Kappa value for consistency between detection methods. With a test level ɑ= 0.05 (two-sided), *P* < 0.05 was considered to indicate statistical significance. The sensitivity was calculated as true positives/(true positives+ false negatives)×100%, and the specificity was calculated as true negatives/(true negatives+ false positives)×100%. The accuracy was computed as (true positives+ true negatives)/(true positives + false negatives+ true negatives+ false positives)×100%.

## 4 Results

### 4.1 Schematic overview of the RT-RPA-PfAgo detection platform for HRSV infection

**Figure 1:**
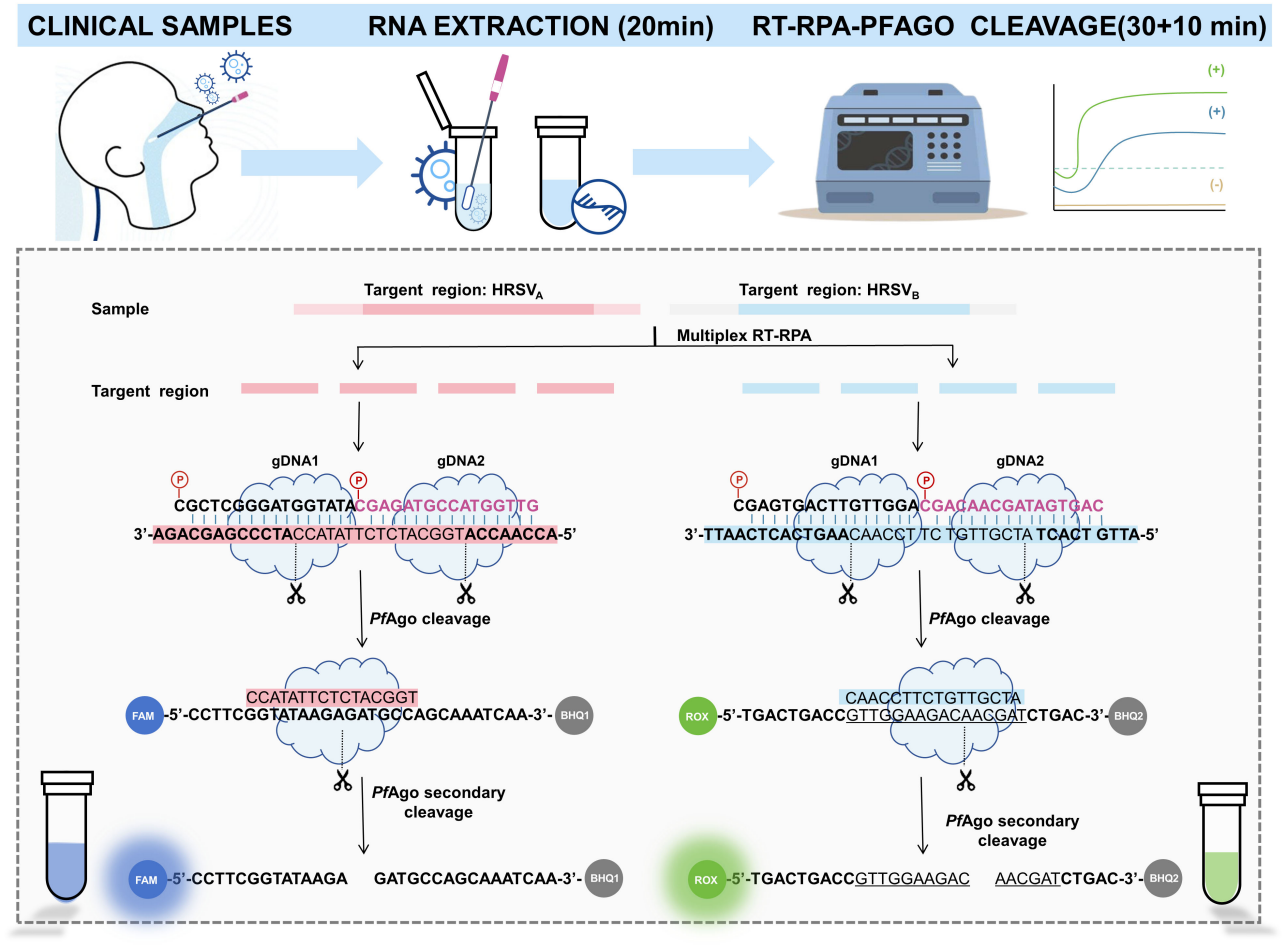
Schematic illustration of the RT-RPA-*PfAgo* platform.

The whole detection process is summarized in **Figure 1**. First, nasopharyngeal swabs were taken from children diagnosed with suspected HRSV infection, and viral genomic RNA was extracted from clinical samples via a commercial RNA extraction kit. Second, the two target regions of HRSV_A_ and HRSV_B_ were simultaneously amplified in one tube by RT-RPA at 39°C. Finally, the purified RPA product is transferred into the *PfAgo* cleavage system, which is followed by incubation at 95°C for 30 min. In the cleavage system, the *PfAgo* protein cleaves the target sequence under the guidance of 5′-phosphorylated gDNA, which results in the formation of new ssDNA. These ssDNAs triggered subsequent *PfAgo* cleavage, targeting specific single-stranded DNA probes with different fluorophore labels and enabling concurrent detection of HRSV_A_ and HRSV_B_ within a single reaction. Only when the probe recognizes the specific single-stranded DNA after the first cleavage will the quenching group and the fluorescent group be separated, thereby generating fluorescence. The quenching group escapes, and the fluorescent group is no longer quenched, and the fluorescence value can finally be read in the real-time fluorescence detector, and the result can be read by the naked eye with the help of ultraviolet light. The entire process can be completed in approximately 1 hour.

### 4.2 Oligonucleotides screening for the RT-RPA-PfAgo detection platform

**Figure 2:**
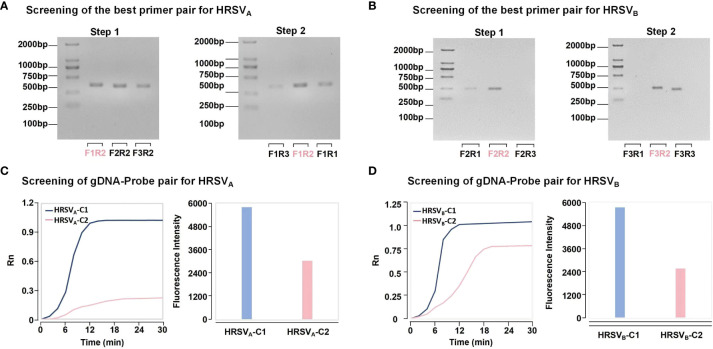
The strategy of best primer pair and gDNA-probe pair screening. **(A)** The strategy for screening the best primer pairs for HRSV_A_. In step 1, a downstream primer was selected to match all the upstream primers. Based on the intensity of the bands, the brightest band is optimal. In step 2, the best upstream primers were used to match all the downstream primers. Finally, the best primer was determined. **(B)** The strategy for screening the best primer pairs for HRSV_B_. **(C)** The strategy for screening the best gDNA-probe pairs for HRSV_A_. The combination of the fastest fluorescence signal growth and the highest final fluorescence intensity was selected to determine the best gDNA-probe pair. HRSV_A_-C1 represents the combination of gDNA1, gDNA2, and probeA1. HRSV_A_-C2 represents the combination of gDNA3, gDNA4 and probeA2. **(D)** The strategy for screening the best gDNA-probe pairs for HRSV_B_. HRSV_B_-C1 represents the combination of gDNA5, gDNA6 and probeB1. HRSV_B_-C2 represents the combination of gDNA7, gDNA8 and probeB2.

Twelve oligonucleotide sequences were designed to screen the best RPA primers for HRSV_A_ and HRSV_B_. The results from TAE gel electrophoresis revealed bands of the expected size, confirming the efficacy of our RPA primer design. Taking HRSV_A_ as an example (**Figure 2A**), one downstream primer, HRSV_A_-R2, was randomly selected to match all the upstream primers (Step 1). Gel electrophoresis revealed that the combination of HRSV_A_-F1/HRSV_A_-R2 had the brightest and clearest band. Therefore, HRSV_A_-F1 was selected as the best upstream primer for combination with all the downstream primers (Step 2). Similarly, the combination of HRSV_A_-F1/HRSV_A_-R2 had a relatively greater amplification efficiency and was confirmed to be the best RPA primer for detecting HRSV_A_. Using the same strategy, we confirmed the best primers for HRSV_B_ (**Figure 2B**). Four gDNA-probe combinations for the detection of HRSV_A_ and HRSV_B_ were designed within the RPA amplification region. As shown in **Figure 2C**, the fluorescence intensity resulting from the combination of HRSV_A_-probeC1 was obviously greater than that resulting from the combination of HRSV_A_-probeC2. Moreover, as shown in **Figure 2D**, the HRSV_B_-probeC1 combination produced greater fluorescence than did the HRSV_B_-probeC2 combination. These results confirmed the best gDNA-probe combinations.

### 4.3 Optimization analysis

**Figure 3:**
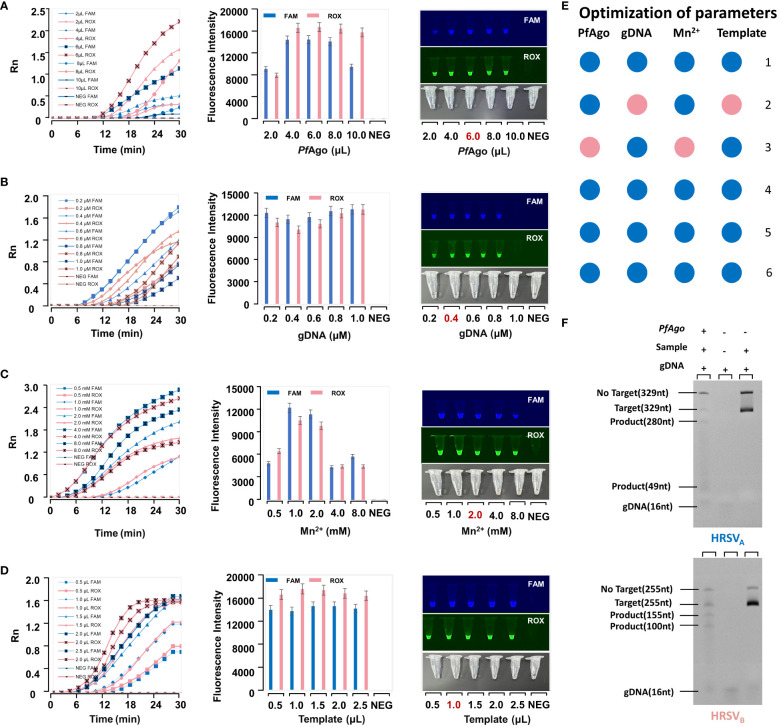
The optimization of parameters in the RT-RPA-*PfAgo* platform. **(A)** Optimization of *Pf*Ago. The optimal condition is the one with the highest fluorescence intensity in the error bar chart. A reaction tube exhibiting fluorescence under UV light is considered positive. **(B)** Optimization of guide DNA. **(C)** Optimization of Mn^2+^. **(D)** Optimization of the template. **(E)** The final reaction system. It is interpreted in conjunction with Figures **(A–D)** The numbers represent the number of tubes. For example, “1” represents 2μL *PfAgo*, 0.2μM gDNA, 0.5μL Mn^2+^, and 0.5μL template. The pink dots were the optimal condition. **(F)** The Urea-PAGE gel electropherogram of RT-RPA-*Pf*Ago cleavage products.

We enhanced the performance of the RT-RPA-*PfAgo* detection platform. *PfAgo* was evaluated at different volumes (2.0, 4.0, 6.0, 8.0, and 10.0 μL) (**Figure 3A**), 6.0 μL was confirmed to be the most effective concentration. Finally, obtaining the most efficient reaction system: 6 μL *PfAgo*, 0.4 μM gDNA, 2.0 mM Mn^2+^, 1.0 μL Template, 2 μL Buffer, 0.4 μM Probe, and add ddH_2_O to a final volume of 25 μL (**Figures 3B–E**).

### 4.4 Validation of the RT-RPA-PfAgo detection platform products

As shown in **Figure 3F**, Lane 1 (from left to right) is the *PfAgo* cleavage product, Lane 2 is gDNA, and Lane 3 is the control. In Lane 1, the target strand (329 nt) formed products of 280 nt and 49 nt in length after *PfAgo* cleavage. Its band in the Urea-PAGE gel was located between the target strand (329 nt) and gDNA (16 nt). The brightness of the target band in Lane 3 was brighter than that in Lane 1. This further demonstrated that *PfAgo* cleaves the target, after which the substrate is consumed.

### 4.5 Sensitivity and specificity

**Figure 4:**
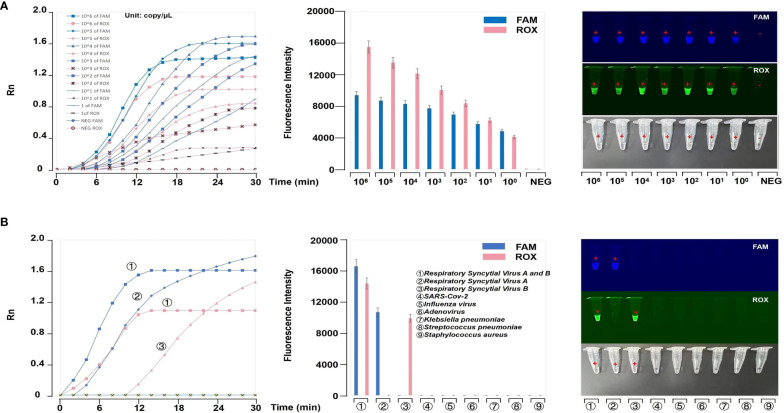
Evaluation of the RT-RPA-*PfAgo* platform. **(A)** Evaluation of the sensitivity of the RT-RPA-*PfAgo* platform. The results showed that the LOD of the platform could reach 1 copy/μL. **(B)** Evaluation of the specificity of the RT-RPA-*PfAgo* platform. The fluorescence curves and images under UV light revealed no positive results for other common respiratory virus samples.

The LODs of both HRSV_A_ and HRSV_B_ reached 1 copy/μL (**Figure 4A**). These results showed that our platform offers high sensitivity for detecting HRSV_A_ and HRSV_B_. Meanwhile, our platform has high specificity (**Figure 4B**). The results were shown by fluorescence curves and were imaged under ultraviolet (UV) light.

### 4.6 Clinical sample analysis

**Figure 5:**
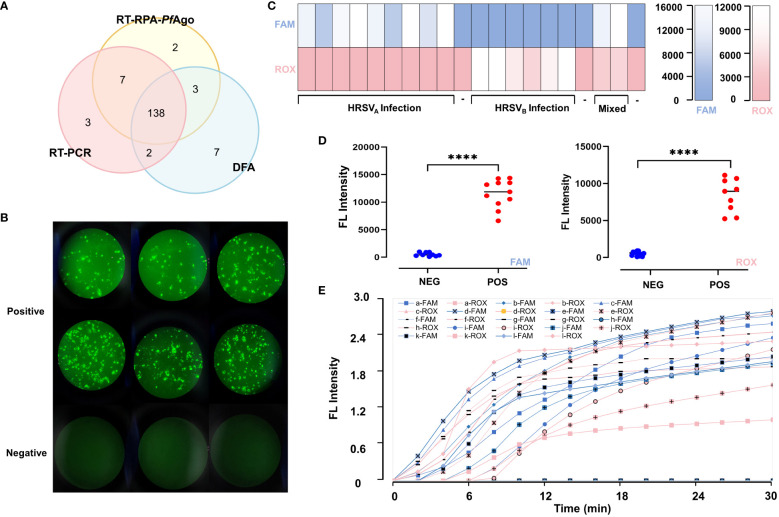
Comparison of the diagnostic capability of the RT-RPA-*PfAgo* platform, RT-PCR and DFA. **(A)** Three methods, including direct immunofluorescence assays, RT-PCR and RT-RPA-*PfAgo* platform, were selected to test 150 clinical samples. TFhe overlapping area represents the number of samples that have the same results across the corresponding detection methods. Compared to DFA, the RT-RPA-*PfAgo* platform is more consistent with RT-PCR. **(B)** Pictures of positive and negative samples from the DFA assays under a fluorescence microscope; no fluorescent signal is seen in the negative results, and cells infected by HRSV are seen in the positive results, with neighboring cells fusing with each other to form syncytial structures **(C)** The fluorescence intensity of twenty clinical samples which were randomly selected. **(D)** The comparison of fluorescence intensity between positive and negative samples for HRSV_A_ and HRSV_A_ infection shows statistically significant differences. **(E)** The fluorescence curve of the 20 random clinical samples. Elevated fluorescence levels in the FAM channel indicate HRSV_A_ infection, whereas elevated ROX fluorescence values indicate HRSV_B_ infection. "****" represents P<0.001.

The extraction products were detected by the RT-RPA-*PfAgo* platform, RT-PCR and DFA (**Figure 5B**). We found our platform is more sensitive than DFA (**Figure 5A**). There were 97 positive samples for HRSV infection, 65 for HRSV_A_ infection, 43 for HRSV_B_ infection and 11 for mixed infection according to the RT-RPA-*PfAgo* platform. We randomly selected 20 patients, and the results were displayed in **Figures 5C–E**. According to some studies, RT-PCR can be regarded as the gold standard. Therefore, we conducted a kappa consistency test for our method compared to RT-PCR. The Kappa coefficient was 0.926 (*P*<0.001), and the accuracy was 96.67%. Our platform displayed an accuracy of 96.0% for HRSV_A_ infection and 97.33% for HRSV_B_ infection. The assay results of our platform were consistent with RT-PCR. The results of the clinical sample assays are shown in **Table 2** and **Supplementary Table S1**.

**Table 2:** Clinical sensitivity, specificity and accuracy of HRSV_A_ and HRSV_B_ genes in clinical sample.

Type of infection	Sensitivity (%)	Specificity (%)	Accuracy (%)
HRSV_A_	92.75% (64/69)	98.76% (80/81)	96.00% (144/150)
HRSV_B_	95.35% (41/43)	98.13% (105/107)	97.33% (146/150)
total	96.00% (96/100)	98.00% (49/50)	96.67% (145/150)

## 5 Discussion

At present, the clinical diagnosis of HRSV infection relies mainly on laboratory indicators, and common methods include virus isolation and culture, antigen detection, nucleic acid detection and serological testing (Goodrich and Miller, 2007; Henrickson and Hall, 2007; Aslanzadeh et al., 2008; Gonzalez and McElvania, 2018). All of these methods have their own advantages and disadvantages (**Supplementary Table S2**). Based on the above data, the RT-RPA-*PfAgo* system has the following advantages. Comparison with traditional methods, in the detection of HRSV, previously reported methods such as ELISA, antigen-antibody detection, etc., the accuracy of their results will be affected by the site of specimen collection, the time of specimen collection and the time from the occurrence of the disease, in the case of the site of specimen collection with low viral concentration and the time from the occurrence of disease to more than 3 days, it may lead to false-negative results. This technology only requires the collection of nasopharyngeal swabs, and can detect HRSV infection at low viral copies. The results could be read by the naked eye within 60 minutes and under ultraviolet light, with less influence by human factors. Compared with the current gold standard method (RT-PCR), real-time fluorescence quantitative detection requires a more complex operation process and complex temperature control equipment, different fluorescence probes and reaction conditions. Different parameters may give different results, but in this experiment, the reaction temperature is relatively single, only the optimal temperature for RPA reaction (39°C) and the temperature required for *PfAgo* to generate cleavage (95°C) were needed, and the final statistics show that the RT-RPA-*PfAgo* system, while possessing the above characteristics, can achieve the same low detection limit as qPCR.

In contrast, the good multiplex ability of *Pf*Ago solves this problem. Unlike PAM-dependent Cas12a, *PfAgo* is guided by two gDNA-guided cuts to produce positive results. This allows the simultaneous presence of multiple fluorescent signals within the result to be interpreted. In the first cleavage, a pair of gDNA is utilized to generate a secondary guide. In the second cleavage, the probe is subjected to a second cleavage in the presence of the secondary guide, resulting in the release of the fluorescent marker (Ye et al., 2022). When multiple targets are present at the same time, multiplex detection is accomplished by their respective labeled fluorescent moieties being different. Therefore, the spiking time, reagent storage conditions, etc., had little effect on the results, and the deviation under the same reaction conditions is likewise negligible. In our study, we identified HRSV_A_ and HRSV_B_ infections simultaneously, which is beneficial for determining the prevalence and dispersion patterns of HRSV and facilitating the development and design of vaccines.

When there is a large-scale epidemic of respiratory infectious diseases with cough, wheezing, and fever as the main clinical manifestations (e.g., Mycoplasma pneumoniae, Streptococcus pneumoniae, whooping cough pneumonia, Hemophilus pneumoniae, adenovirus pneumoniae, and influenza viruses, etc.), antibiotic misuse is likely to occur in some primary care hospitals or among inexperienced healthcare professionals when dealing with these patients or may result in inappropriate decisions in patient management. For example, if a child with HRSV infection develops fever, cough, or wheezing, and when there is no specific change in the results of blood counts and inflammation indexes, the child may be misdiagnosed with pertussis pneumonia and prophylactically use macrolide antibiotics to counter the infection, or co-locate the HRSV infection with a child infected by Bordetella pertussis, which may lead to worsening of wheezing, prolongation of the course of the disease, or even death.

From this point of view, the detection of pathogens within a short period of time after admission to the hospital is particularly critical, on the one hand, doctors and nurses can make appropriate anti-infective treatment according to the pathogens, thereby reducing the abuse of antibiotics and reducing the emergence of antibiotic resistance. On the other hand, from the point of view of nosocomial infection patient management, placing patients with the same pathogen in the same space can greatly reduce the occurrence of cross-infection, thus shortening the length of hospitalization of children and reducing the consumption of unnecessary medical resources. At the same time, in areas lacking specialized testing instruments or professional technicians, the relatively simple experimental conditions and operating procedures also promote the development of POC technology in these areas.

Regarding vaccine development, there is currently no HRSV vaccine that is actually used in clinics. This is because infants infected with HRSV are younger and require vaccination at birth or shortly after birth due to a number of factors, such as the infant’s immature cellular and humoral immune system or the presence of antibodies derived from their mother. As a result, their response to the vaccine may differ significantly from that of infants who are not infected with HRSV (Griffiths et al., 2017). Furthermore, as newborns have received multiple vaccinations on a regular basis, it is important to ensure that the HRSV vaccine does not compromise the effectiveness of these other vaccinations, and vice versa. Therefore, the purpose of this experiment is to support the development of an RSV vaccine by creating molecular diagnostic assays that can be used for both the quick detection of HRSV and the typing of HRSV subpopulations. Additionally, future experiments will be able to validate multiple HRSV genotypic subtype infections in a single tube by using different HRSV genotypes as detection targets.

Finally, we evaluated the usability of the platform. The method is easy to perform and does not require operation on ice. No complex temperature conditions are required for the reaction to proceed, which significantly enhances the operability of the platform. Therefore, this technique can complete pathogen detection in environments where professional equipment is lacking or rudimentary. Meanwhile, obtaining accurate results within approximately 1 hour is crucial for improving efficiency and reducing waiting time in clinical interventions. Moreover, in terms of cost, the reagents and consumables needed for each experiment were only $4.3. Because of its low cost, this type of method makes large-scale POC testing possible when there is a large-scale HRSV infection outbreak, providing new ideas for infection prevention and control and preventing the abuse of antibiotics.

## 6 Conclusion

In conclusion, this study constructed the RT-RPA-*Pf*Ago platform for HRSV, which has high sensitivity, great specificity and high accuracy. This POC testing facilitates easier detection of HRSV infections in regions with limited resources and inadequate expertise, assisting healthcare professionals in effective management and treatment and ultimately contributing to better patient outcomes and reducing the burden on healthcare systems.

## Data availability statement

The raw data supporting the conclusions of this article will be made available by the authors, without undue reservation.

## Ethics statement

The studies involving humans were approved by The Ethics Committee of the Second Affiliated Hospital of Shantou University Medical College. The studies were conducted in accordance with the local legislation and institutional requirements. Written informed consent for participation in this study was provided by the participants’ legal guardians/next of kin.

## Author contributions

JL: Methodology, Writing – original draft. XF: Writing – review & editing. JZ: Writing – original draft. TY: Software, Validation, Writing – original draft. MZ: Software, Writing – original draft. YZ: Methodology, Writing – original draft. WH: Writing – original draft. HL: Software, Writing – original draft. LK: Writing – review & editing. SC: Writing – original draft. NZ: Writing – original draft. JF: Writing – original draft. XC: Writing – review & editing. JC: Writing – original draft. GL: Writing – review & editing. LL: Writing – review & editing. WC: Writing – review & editing. YL: Writing – original draft. FH: Writing – original draft. CL: Funding acquisition, Resources, Writing – review & editing. ML: Conceptualization, Writing – review & editing.
