# Supplementary material for: RT-RPA-PfAgo detection platform for one-tube simultaneous typing diagnosis of human respiratory syncytial virus
Source: Front Cell Infect Microbiol. 2024 Jul 25;14:1419949. doi: 10.3389/fcimb.2024.1419949 (PMC11306018; doi:10.3389/fcimb.2024.1419949)
Supplement: Supplementary file 1 [file Table_1.docx]

**Tab. S1 The clinical evaluation of 150 sample.**

| RSV_A_ | | RT-PCR | |  | Kappa | *P* |
| --- | --- | --- | --- | --- | --- | --- |
|  |  | positive | negative | total |  |  |
| RT-RPA-PfAgo | positive | 64 | 1 | 65 | 0.919 | ＜0.001 |
|  | negative | 5 | 80 | 84 |  |  |
|  | total | 69 | 81 | 150 |  |  |
| RSV_B_ | | RT-PCR | |  | Kappa | *P* |
|  |  | positive | negative | total |  |  |
| RT-RPA-PfAgo | positive | 41 | 2 | 43 | 0.935 | ＜0.001 |
|  | negative | 2 | 105 | 108 |  |  |
|  | total | 43 | 107 | 150 |  |  |

| Total | | RT-PCR | |  | Kappa | *P* |
| --- | --- | --- | --- | --- | --- | --- |
|  |  | positive | negative | total |  |  |
| RT-RPA-PfAgo | positive | 96 | 1 | 97 | 0.926 | ＜0.001 |
|  | negative | 4 | 49 | 53 |  |  |
|  | total | 100 | 50 | 150 |  |  |
