# Supplementary material for: RT-RPA-PfAgo detection platform for one-tube simultaneous typing diagnosis of human respiratory syncytial virus
Source: Front Cell Infect Microbiol. 2024 Jul 25;14:1419949. doi: 10.3389/fcimb.2024.1419949 (PMC11306018; doi:10.3389/fcimb.2024.1419949)
Supplement: Supplementary file 2 [file Table_2.docx]

**Table S2 Advantages and disadvantages of several clinical diagnostic methods for HRSV infection**

| **Method** | **Advantages** | **Disadvantages** | **Time** |
| --- | --- | --- | --- |
| Virus isolation and culture | Gold standard for laboratory diagnosis with high specificity | It is time-consuming and requires high operating and laboratory environmental conditions, and is no longer suitable for clinical laboratory diagnosis | 3~14 days |
| RT-qPCR | High sensitivity, excellent specificity, can detect multiple pathogens nucleic acid at the same time | SNPs and other mutations can lead to false negative results. Analysis of results when multiple pathogens are positive should be combined with clinical | 2~8 hours |
| Rapid antigen detection | Results can be obtained within 30 minutes, easy to operate, and applicable to POCT | Low sensitivity, only one sample can be read at a time | 30 mins |
| Direct immunofluorescence assay(DFA) | Good sensitivity and specificity | Sufficient respiratory columnar epithelial cells are required in the sample and high demands on operation | 3~4 hours |
